# ﻿Unveiling new species of Phragmidiaceae (Basidiomycota, Pucciniales) on rosaceous plants from Guizhou, China

**DOI:** 10.3897/mycokeys.115.146604

**Published:** 2025-03-28

**Authors:** Qinfang Zhang, Qianzhen Wu, Peng Zhao, Kamran Habib, Yao Wang, Dexiang Tang, Muhammad AIjaz Ahmad, Yulin Ren, Xiangchun Shen, Qingde Long, Lili Liu, Qirui Li

**Affiliations:** 1 State Key Laboratory of Discovery and Utilization of Functional Components in Traditional Chinese Medicine & School of Pharmaceutical Sciences, Guizhou Medical University, Guian New District, Guizhou 550004, China Guizhou Medical University Guizhou China; 2 The High Efﬁcacy Application of Natural Medicinal Resources Engineering Center of Guizhou Province (The Key Laboratory of Optimal Utilization of Natural Medicine Resources), School of Pharmaceutical Sciences, Guizhou Medical University, University Town, Guian New District, Guizhou 550004, China Institute of Microbiology, Chinese Academy of Sciences Beijing China; 3 State Key Laboratory of Mycology, Institute of Microbiology, Chinese Academy of Sciences (CAS), Beijing 100101, China Guizhou Medical University Guizhou China; 4 Immune Cells and Antibody Engineering Research Center of Guizhou Province, Guizhou Medical University, Guiyang 550004, China Institute of Microbiology, Chinese Academy of Sciences Beijing China

**Keywords:** ITS, LSU, Phragmidiaceae, phylogeny, rust disease, taxonomy

## Abstract

Rust fungi associated with *Rubus* were collected across diverse locations in Guizhou Province, and three new species – *Gerwasiaamphidasydis* on *Rubusamphidasys*, *Phragmidiumcoreanicola* on *Rubuscoreanus*, and *Phragmidiumparvifolius* on *Rubusparvifolius* are introduced. These novel species are described based on morphological characteristics and phylogenetic analysis of the ITS and LSU loci. Additionally, *Gerwasiarubi-setchuenensis* is introduced as a new host record on *Rubusbuergeri*. The study includes comprehensive morpho-anatomical descriptions, detailed illustrations, and a phylogenetic tree, providing insights into the taxonomic placement and relationships of these novel taxa within their respective lineages.

## ﻿Introduction

Pucciniales (Basidiomycota, Pucciniomycetes) represents approximately 25% of basidiomycete fungi and constitutes one of the most prevalent fungal groups, parasitizing leaves, fruits, and branches of plants, thereby inhibiting normal growth and development of plants and impacting yield and quality (Aime and McTaggart 2021; Zhao et al. 2022a). To date, seven suborders (i.e., Araucariomycetineae, Melampsorineae, Mikronegeriineae, Raveneliineae, Rogerpetersoniineae, Skierkineae, and Uredinineae) and 18 families (i.e., Araucariomycetaceae, Coleosporiaceae, Crossopsoraceae, Gymnosporangiaceae, Melampsoraceae, Milesinaceae, Ochropsoraceae, Phakopsoraceae, Phragmidiaceae, Pileolariaceae, Pucciniaceae, Pucciniastraceae, Raveneliaceae, Rogerpetersoniaceae, Skierkaceae, Sphaerophragmiaceae, Tranzscheliaceae, and Zaghouaniaceae) have been reported (Aime et al. 2017; Aime and McTaggart 2021; Zhao et al. 2022b).

According to estimations, there are approximately 35,000 higher plant species in China, which are categorized into 454 families and 3,818 genera (Wang et al. 2015). It is further estimated that between 1,700 and 8,800 species of rust fungi may exist within the country, posing a significant threat to the health and productivity of these plant species (Zhao et al. 2021). More than 8,400 rust taxa have been documented worldwide (Zhao et al. 2021; Sun et al. 2022). Based on their morphological characteristics and host associations, 1,200 species from 71 genera across 15 families have been documented in China. Among them, more than 70 species of *Phragmidium* have been identified. (Dai 1979; Zhuang et al. 1998, 2003, 2005, 2012, 2021; Ji et al. 2019, 2020; Zhao et al. 2021; Sun et al. 2022, 2024). Plants are susceptible to Coleosporiaceae, Melampsoraceae, Phragmidiaceae, and Pucciniaceae, which are the dominant families of rust taxa in China (Zhao et al. 2021; Zhao et al. 2022b; Sun et al. 2024).

The genus *Phragmidium* (Pucciniales: Phragmidiaceae) was established by Link (1816). Phragmidiaceae comprises a diverse array of species, encompassing 14 genera and an estimated 200 species, primarily targeting the economically vital family Rosaceae (examples of hosts in Rosaceae: *Rosa*, *Rubus*, *Potentilla*, *Sanguisorba*, *Duchesnea*, and *Acaena*) (Zhao et al. 2021; Sun et al. 2024). A total of 115 *Phragmidium* species have been described (Cummins 1931; Arthur 1934; Wahyuno et al. 2001; Cummins and Hiratsuka 2003; Zhuang et al. 2012; Yang et al. 2015; Ali et al. 2017; Zhao et al. 2021; Sun et al. 2022; Ya 2024). Among these, 38 species specifically target and parasitize members of the genus *Rosa* (Ya 2024), and at least 39 *Phragmidium* species have been reported in China (Wei 1988; Zhuang et al. 2003, 2012; Yang et al. 2015; Liu et al. 2018).

The susceptibility of Rosaceae plants to Phragmidiaceae infections underscores the importance of understanding the biology, ecology, and management strategies of these fungal pathogens (Dietel 1905; Yun et al. 2011; Sun et al. 2022). During the field investigation of rust fungi on medicinal plants in Guizhou Province, China, three potential new species were found belonging to the genera *Gerwasia* and *Phragmidium* of Phragmidiaceae, which infect the members of Rosaceae, as well as one species that infects a new host. We conducted a phylogenetic analysis using multi-locus (ITS and LSU) phylogeny and morphological characteristics to better understand their taxonomic position. Descriptions, illustrations, and phylogenetic analysis results of all the novel species and the new host record are provided.

## ﻿Materials and methods

### ﻿Sample collection and preservation

Rust-infected specimens were collected in Guizhou Province, China, from August to November of each year from 2021 to 2023. All host and habitat information of specimens was recorded (Rathnayaka et al. 2024). Photographs of the infected plants were taken using a camera (Canon G15, Corporation, Tokyo, Japan). The samples were kept in blotting papers and were brought to the laboratory for examination. The collected specimens were partly kept in a refrigerator at 4 °C for spare parts and partly pressed and air-dried to make pressed specimens (Wei and Wang 2011; Wu et al. 2023). All specimens were deposited at the herbarium of Guizhou Medical University (**GMB**) and Kunming Institute of Botany, Chinese Academy of Sciences (**KUN-HKAS**).

### ﻿Morphological characterization

Macroscopic characteristics were observed under a stereomicroscope (Olympus SZ61), and photographs were taken with a digital camera (Canon 700D) fitted with a light microscope (Nikon Ni). The infected portions were examined, and photographs were taken as described by Wu et al. (2023). More than 30 measurements were noted for each type of teliospores, urediniospores, and paraphyses for each sample using the Tarosoft (R) Image Frame Work v. 0.9.0.7. The images were arranged using Adobe Photoshop CS6 (Adobe Systems, the USA).

### ﻿DNA extraction and PCR amplification

The infected portion of the rust fungus was scraped using a sterilized scalpel. DNA extraction was carried out following the manufacturer’s protocols for the Biomiga Fungal gDNA Kit. The DNA samples were kept at –20 °C. The rust-specific primer pairs Rust2inv (Aime 2006) and ITS4rust (Pfunder et al. 2001) were used for the PCR amplification of the regions of internal transcribed spacer (ITS), whereas the universal primer pairs LR0R (Hopple and Vilgalys 1999) and LR6 (Vilgalys and Hester 1990) were used for the large subunit ribosomal (LSU). The composition of a 25 μL PCR mixture comprised the following: 9.5 μL of double-distilled water, 12.5 μL of PCR Master Mix, 1 μL of each primer, as well as 1 μL of template DNA. The qualified PCR products were verified through 1.5% agarose gel electrophoresis, stained with GoldenView, and subsequently submitted to Sangon Co. China for sequencing (Zhao et al. 2016).

### ﻿Phylogenetic analyses

All sequences were obtained in ABI file format and deposited in the GenBank (Table 1). The consensus sequences were blasted in GenBank using the BLAST algorithm. The similar sequences were retrieved from the GenBank database. The molecular phylogeny was inferred from a combined dataset of ITS and LSU sequences. The reference sequences retrieved from open databases originated from recently published literature (Zhao et al. 2021; Wu et al. 2023; Sun et al. 2024). All the ambiguous nucleotides were trimmed using BioEdit software v.7.0.5.3 and TrimAL (Hall 1999). Sequences were aligned using the MAFFT v.7.110 online tool (Katoh and Standley 2013). The alignments are available in TreeBASE (www.treebase.org/treebase-web/home.html) under ID31754 for LSU and ITS rDNA sequences. The maximum likelihood (ML) analysis was implemented in RAxML v.8.2.12 using the GTRGAMMA substitution model with 1,000 bootstrap replicates (Stamatakis 2015). The phylogenetic analyses were also performed using Bayesian inference in MrBayes v.3.2.1 (Ronquist et al. 2015) online. The Markov chain Monte Carlo (MCMC) sampling in MrBayes v.3.2.2 (Ronquist et al. 2015) was used to determine the Bayesian posterior probabilities (BYPP). Six simultaneous Markov chains were run for 3,000,000 generations, and trees were sampled every 1,000^th^ generation. All analyses were run on the CIPRES Science Gateway v.3.3 web portal (Miller et al. 2010; Huelsenbeck and Ronquist 2001). The phylogenetic tree was visualized by FigTree v.1.4.3 (Rambaut 2012).

**Table 1.** Taxa information and corresponding GenBank accession numbers of the sequences used in the phylogenetic analyses.

Species	Specimen No.	Host	Province, Country	GenBank Accession No.		References
				ITS	LSU	
*Gerwasiachinensis*	HMAS249978 (ZP-R5)	*Rubusparkeri*	Yunnan, China	MK519039	MK518737	Zhao et al. 2021
*G.chinensis*	HMAS249980 (ZP-R295)	*Rubusparkeri*	Yunnan, China	MK519038	MK518540	Zhao et al. 2021
*G.pittieriana*	BPI 843556	*Rubus* sp.	USA	KY764065	KY764065	Zhao et al. 2021
*G.rubi*	(ZP-R345)	*Rubussetchuenensis*	Sichuan, China	–	MK518735	Zhao et al. 2021
*G.amphidasydis*	GMB4047*	*Rubusamphidasys*	Guizhou, China	PQ472136	PQ456450	This study
*G.amphidasydis*	GMB4076	*Rubusamphidasys*	Guizhou, China	PQ472137	PQ456451	This study
*G.rubi-setchuenensise*	GMB4052	*Rubusbuergeri*	Guizhou, China	PQ472135	PQ456449	This study
*G.rubi-setchuenensise*	GMB4075	*Rubusbuergeri*	Guizhou, China	PQ472134	PQ456448	This study
*G.rubi-setchuenensis*	HGUP21168*	*Rubussetchuenensis*	Guizhou, China	OR470045	OR528540	Sun et al. 2024
*G.rubus-playfairianus*	HMAS249840 (ZP-R 1374)	*Rubusplayfairianus*	Guangxi, China	MK518976	MK518674	Sun et al. 2024
*Gymnosporangiumasiaticum*	CUP-0016*	*Juniperuschinensis*	Japan	MN642593	MN642617	Zhao et al. 2020
*Gymnosporangiumsabinae*	TNM F0030477	*Pyruscommunis*	Bulgaria: Sofia	KY964764	KY964764	Shen et al. 2017
*Hamasporaacutissima*	BRIP:55606	*Rubusrolfei*	Philippines	–	KT199398	McTaggart et al. 2016
*H.sinica*	ZP-R1	*Rubussetchuenensis*	Guangdong, China	MK519049	MK518636	Zhao et al. 2020
*H.longissima*	BPI 871506	*Rubusrigidu*	South Africa	–	MW049262	Aime and McTaggart 2021
*H.rubi-lambertianuse*	HGUP21164*	*Rubuslambertianus*	Guizhou, China	OR470053	OR528547	Sun et al. 2024
*H.rubi-parkerii*	HGUP21159*	*Rubusparkeri*	Guizhou, China	OR470055	OR528543	Sun et al. 2024
*H.rubi-alceifolii*	GMB0109*	*Rubusalceifolius*	Guizhou, China	OQ067094	OQ067532	Wu et al. 2023
*H.rubi-alceifolii*	GMB0116	*Rubusalceaefolius*	Guizhou, China	OQ067095	OQ067533	Wu et al. 2023
*Kuehneolauredinis*	LD1029	*Rubus* sp.	New York, America	GU058013	GU058013	Dixon et al. 2018
*Phragmidiumbarnardii*	HGUP21035	*Rubusparvifolius*	Guizhou, China	OL684828	OL684839	Sun et al. 2022
*P.charyuensis*	BJFC: R02532*	*Rosaduplicata*	China	MH128374	NG_064492	Liu et al. 2018
*P.cibanum*	BJFC: R02528	*Rubusniceus*	China	MH128370	NG_064491	Liu et al. 2018
*P.cymosum*	GMB0115	*Rosacymosa*	Guizhou, China	OQ067097	OQ067531	Wu et al. 2023
*P.cymosum*	GMB0108*	*Rosacymosa*	Guizhou, China	OQ067096	OQ067530	Wu et al. 2023
*P.coreanicola*	GMB0101*	*Rubuscoreanus*	Guizhou, China	PQ472133	PQ456447	This study
*P.coreanicola*	GMB4071	*Rubuscoreanus*	Guizhou, China	PQ472132	PQ456448	This study
*P.griseum*	HMAS56906	*Rubuscrataegifoliu*	Beijing, China	MH128377	MG669115	Liu et al. 2018
*P.griseum*	BJFCR 03451	–	Beijing, China	MN264713	MN264731	Liu et al. 2020
*P.griseum*	BJFCR03449	*Rubuscrataegifoliu*	Beijing, China	MN264712	MN264730	Liu et al. 2020
*P.japonicum*	HMAS41585	*Rosalaecigata*	Fujian, China	MN264716	MN264734	Liu et al. 2020
*P.jiangxiense*	BJFCR 03453*	*Rosalaecigata*	Jiangxi, China	MN264715	MN264733	Liu et al. 2020
*P.kanasense*	ZP-R1382	*Phoenixacaulis*	Yunnan, China	MK518980	MK518678	Zhao et al. 2021
*P.kanasense*	ZP-R491	*Rosafedtschenkoana*	Xinjiang, China	–	MK518748	Zhao et al. 2021
*P.leucoaecium*	BJFCR 02118*	*Rosa* sp.	Yunnan, China	MN264719	MN264737	Liu et al. 2020
*P.mexicanum*	E14_5_1	*Potentillaindica*	Slovenia, Forestry	LN795901	LN795901	Piškur and Jurc 2017
*P.octoloculare*	HMAS140416	*Rubusbiflorus*	China	MH128376	MG669119	Liu et al. 2018
*P.pauciloculare*	ZP-R318	*Rubuscorchorifolius*	Guangxi, China	MK518874	MK518542	Zhao et al. 2021
*P.potentillae*	HGUP21034	*Acaenanovae-zelandiae*	Tasmania, Australia	OL684827	OL684838	Sun et al. 2022
*P.potentillae*	HMJAU8609	*Potentillachinensis*	China	MK296538	MK296520	Ji et al. 2019
*P.potentillae*	BJFCR 00961	*Potentillachinensis*	Beijing, China	MN264720	MN264738	Liu et al. 2020
*P.potentillae*	TJ-1F	*Potentillachinensis*	China	PP272995	PP266810	Liu et al. 2020
*P.potentillae*	GMB4048	*Potentillachinensis*	Guizhou, China	PQ472142	PQ456456	This study
*P.potentillae*	GMB4072	*Potentillachinensis*.	Guizhou, China	PQ472143	PQ456457	This study
*P.rosae-multiflorae*	HGUP21158	*Rosamultiflora*	Guizhou, China	OR470059	OR528548	Sun et al. 2024
*P.rosae-multiflorae*	BIFCR 03454	*Rosamultiflora*	Jiangxi, China	MN264721	MN264739	Liu et al. 2020
*P.rosae-multiflorae*	HMAS94924	*Rosamultiflora*	Zhejiang, China	–	KU059175	Sun et al. 2022
*P.rosae-multiflorae*	GMB4044	*Rosamultiflora*	Guizhou, China	PQ472144	PQ456459	This study
*P.rosae-multiflorae*	GMB4073	*Rosamultiflora*	Guizhou, China	PQ472145	PQ456458	This study
*P.rosae-roxburghii*	HGUP21025*	*Rosaroxburghii*	Guizhou, China	OL684818	OL684831	Liu et al. 2018
*P.rosae-roxburghii*	GMB0104	*Rosaxanthina*	Guizhou, China	OQ067092	–	Wu et al. 2023
*P.rosae-kwangtungensise*	HGUP21154*	*Rosakwangtungensis*	Guizhou, China	OR470067	–	Sun et al. 2024
*P.rosae-cymosaii*	HGUP21147	*Rosacymosa*	Guizhou, China	OR470062	OR528551	Sun et al. 2024
*P.rubi-idaei*	HMUT100470	*Rubussaxatilis*	Chongqing, China	OQ613354	OQ606768	–
*P.rubi-oldhami*	KSNUH1322	*Rubuspungens*	South Korea	ON180674	ON170371	Kim et al. 2022
*Phragmidium* sp.	HMJAU8613	–	Changchun, China	MK398297	MK398296	Ji et al. 2019
*P.parvifolius*	GMB4054*	*Rubusparvifolius*	Guizhou, China	PQ472140	PQ456454	This study
*P.parvifolius*	GMB4070	*Rubusparvifolius*	Guizhou, China	PQ472141	PQ456455	This study
*P.tormentillae*	GMB00114	*Potentillasimulatrix*	Guizhou, China	OQ067093	–	Wu et al. 2023
*P.tormentillae*	BPI 843392	*Duchesnea* sp.	USA: Maryland	–	DQ354553	Yun et al. 2011
*P.violaceum*	KRM 0035511	*Rubus* sp.	Germany	ON063390	ON063390	Bradshaw et al. 2023
*P.violaceum*	ZP-R1384	*Duchesneaindica*	Yunnan, China	MK518982	MK518680	Zhao et al. 2021
*P.yangii*	BJFCR 00338	*Rosalichiangensis*	Beijing, China	MN264725	MN264743	Yang et al. 2015
*P.zangdongi*	BJFC: R02447*	*Rosatibetica*	Tibet, China	MH128372	NG064490	Liu et al. 2018
*P.zhouquensis*	BJFCR01516	*Rosaomeiensis*	Yunnan, China	MN264728	MN264746	Yang et al. 2015
*P.zhouquensis*	BJFCR01529	*Rosaomeiensis*	Yunnan, China	MN264729	MN264747	Liu et al. 2020
*Trachysporaalchemillae*	BPI 843828	*Alchemillavulgari*	Switzerland	DQ354550	DQ354550	Aime 2006

Notes: –: no data available; *: type specimens or strains.

## ﻿Results

### ﻿Phylogeny

In this study, 14 samples from twelve host plant species were collected in Guizhou Province. Through morphological and molecular systematic studies, a total of 6 species were identified, including three new species, one new host species, and two known species.

For the final phylogenetic analyses, taxa were selected based on their morphological and phylogenetic affinities, largely following the approach of Zhao et al. (2021). Both the RAxML and BYPP analyses produced similar overall tree topologies with no significant differences. The alignment includes 67 species (comprising one family: Phragmidiaceae, six genera: *Phragmidium*, *Gerwasia*, *Hamaspora*, *Kuehneola*, *Trachyspora*, and the outgroup genus *Gymnosporangium*) and contains 1245 characters, including gaps (ITS: 398 bp, LSU: 847 bp). Among these, three novel species have been identified (*Gerwasiaamphidasydis* sp. nov., *Phragmidiumparvifolius* sp. nov., and *P.coreanicola* sp. nov.). Additionally, one species (*G.rubi-setchuenensise*) was reported for the first time on *Rubusbuergeri*.

### ﻿Taxonomy

#### ﻿Family Phragmidiaceae Corda, Icon. Fung. (Prague) 1: 6. 1837

**Genus *Gerwasia* Racib. Bull. Acad. Sci. Lett. Cracovie, Cl. Sci. Math. Nat. Sér. B, Sci. Nat. 3: 270. 1909**

##### ﻿ Gerwasia amphidasydis

Taxon classificationFungiPuccinialesPhragmidiaceae

Q. F. Zhang, Q. Z. Wu & Q. R. Li sp. nov.

72625E3E-7BB9-51D7-8BF5-55B5325D1A33

854995

Fig. 2

###### Type.

China • Guizhou Province, Zunyi City, Kuangkuoshui Nature Reserve (28°12'40"N, 107°10'22"E), 2227 m a.s.l., on leaves of *Rubusamphidasys*. 1 November 2022, Q. Z. Wu and Q. F. Zhang (holotype GMB4047, isotype KUN-HKAS144247);

###### Etymology.

The epithet refers to the host species, *Rubusamphidasys* Focke ex Diels., from which the holotype was collected.

###### Description.

***Spermogonia***, ***Aecia***, and ***Telia*** not found. ***Uredinia*** 0.2–0.8 mm diam. produced on the abaxial leaf surface, scattered to gregarious, hypophyllous, covered by peridium, small, rounded, light yellow, or orange-yellow. Urediniospores 29–41 × 22–29 μm (av. = 34 × 26 μm, n = 30), globose to subglobose or ovoid, golden, yellow-brown, wall 1.2–2.5 µm thick at sides, hyaline, prominent sparsely echinulate, markings elongated longitudinally, 1.3–3.1 µm in distance, pore obscure, germ pores inconspicuous. Pedicel broken; paraphyses not seen.

###### Additional material examined.

China • Guizhou Province, Zunyi City, Kuangkuoshui Nature Reserve, (28°12'38"N, 107°10'21"E) 2214 m a.s.l., on the leaves of *Rubusamphidasys* (Rosaceae). 1 November 2022, Q. Z. Wu and Q. F. Zhang (GMB4076).

###### Notes.

**Figure 1.**
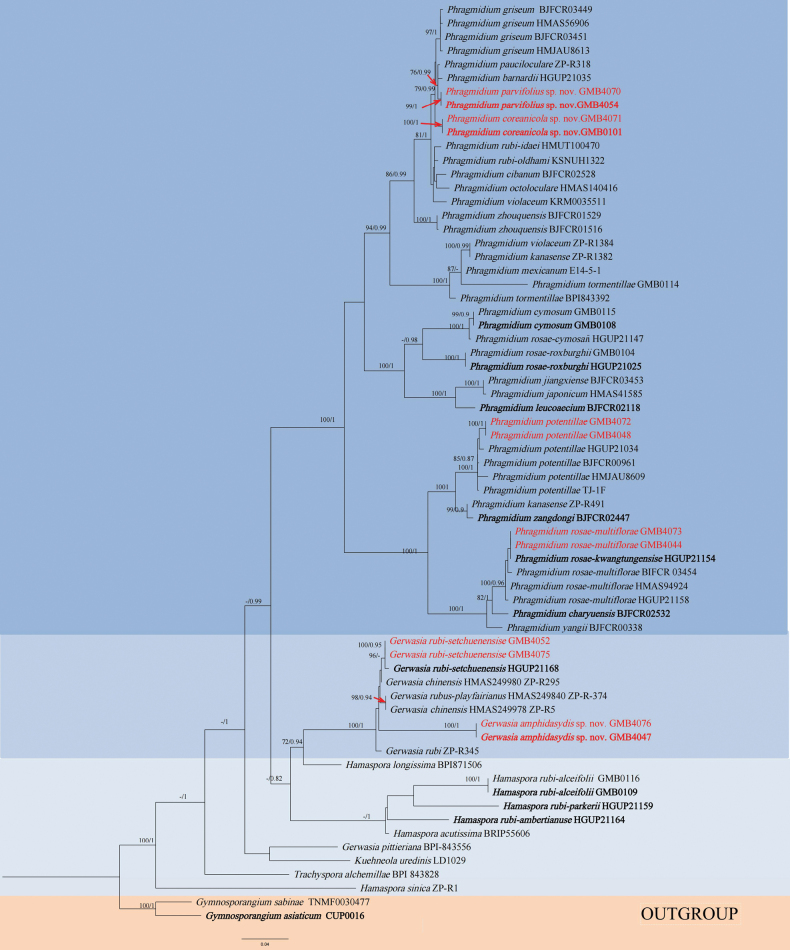
RAxML tree of the family Phragmidiaceae based on rDNA ITS and LSU sequences. ML bootstrap supports (≥75%) and Bayesian posterior probability (≥0.90) are indicated as ML/BYPP. The tree is rooted to *G.sabinae* and *G.asiaticum* (Yang et al. 2015; Aime et al. 2018). All species newly studied are indicated in red, with novel species highlighted in bold red. Type materials were highlighted in bold.

*Gerwasiaamphidasydis* was the first species of *Gerwasia* described on *Rubusamphidasys*. Our phylogenetic analyses showed that *G.amphidasydis* formed a separate branch (Fig. 1). Morphologically, *G.amphidasydis* and *G.rubi* exhibit similar spines. Moreover, the difference between *G.amphidasydis* and *G.rubi* is that the former has bigger urediniospores (29–41 × 22–29 μm vs. 22–33 × 16–26 µm) (Ito 1950; Hiratsuka et al. 1992). *Gerwasiaamphidasydis* and *G.guanganensis* have similar *uredinia* and *urediniospores*; however, *G.guanganensis* has longer spine distances compared to *G.amphidasydis* (4.0–6.0 µm vs. 1.3–3.1 µm) (Zhao et al. 2021). *Gerwasiaamphidasydis* is distinguishable from *G.rubi-setchuenensise* by having larger urediniospores (29–41 × 22–29 μm vs. 18–29 × 15–22 μm) and a thinner wall (1.2–2.5 µm vs. 2.1–3.2 μm) (Sun et al. 2024).

**Figure 2.**
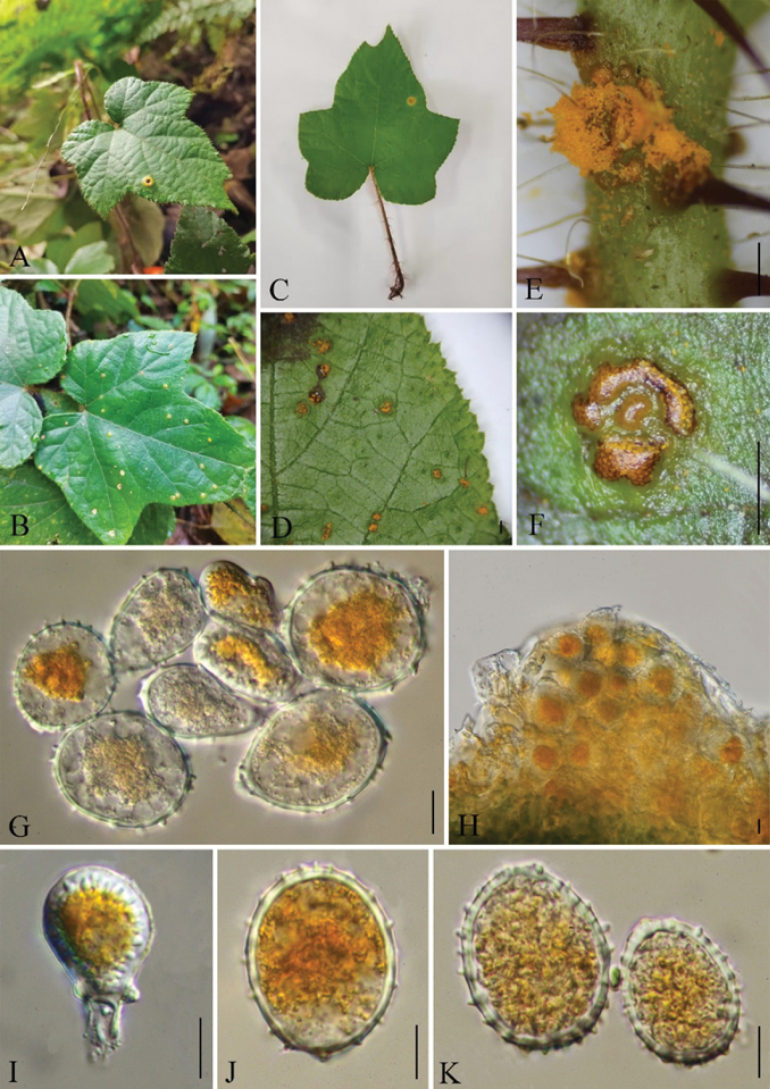
*Gerwasiaamphidasydis* (Holotype GMB4047) **A–D** host and its habitat **E–F** uredinia under a stereomicroscope **G–K** urediniospores. Scale bars: 1 mm (**D, F)**; 0.5 mm **(E)**; 10 μm (**G–K)**.

Additionally, the LSU sequences of *Gerwasiaamphidasydis* also differ from that of *G.rubi* with 93.74% similarity and from *G.rubi-setchuenensis* with 90.56% similarity. The ITS sequence for *Gerwasiarubi* is not available in the NCBI database, whereas the ITS sequence similarity between *Gerwasiaamphidasydis* and *G.rubi-setchuenensis* is 98.56%.

#### ﻿Genus *Phragmidium* Link, Mag. Gesell. Naturf. Freunde, Berlin 7: 30. 1816

##### ﻿ Phragmidium coreanicola

Taxon classificationFungiPuccinialesPhragmidiaceae

Q. F. Zhang, Q. Z. Wu & Q. R. Li sp. nov.

0E3E9EE1-38C7-59E6-A93F-00A48BC63344

855005

Fig. 3

###### Type.

China • Guizhou Province, Guiyang City, Campus of Guizhou Medical University (26°22'48"N, 106°37'30"E), 1911 m a.s.l., on leaves of *Rubuscoreanus* (Rosaceae), 7 October 2021, Q. Z. Wu (holotype GMB0101, isotype KUN-HKAS144249).

###### Etymology.

The epithet refers to the host species, RubuscoreanusMiq.var.coreanus, from which the holotype was collected.

###### Description.

***Spermogonia*** and ***Aecia*** not found. ***Uredinia*** 0.1–0.7 mm diam., produced on the abaxial leaf surface, scattered to gregarious, hypophyllous, yellow spots, scattered, irregular patches. Urediniospores 20–29 × 14–25 μm (av. = 24 × 21 μm, n = 30), globose to subglobose or broadly elliptical to ellipsoidal, wall 0.8–2.1 μm thick (av. = 1.4 μm, n = 30), inconspicuous or smooth at the base; inclusions orange-yellow or pale-yellow; germ pores 2–3, sub-equatorial. ***Telia*** 0.1–0.9 mm diam., hypophyllous, dark brown to black, clustered or scattered, bacilliform. Teliospores 107–167 × 25–35 μm (av. = 134 × 30 μm, n = 30), cylindrical, 5–7 cells, often 6, reddish-brown to opaque, rounded at the apex, rounded or somewhat attenuate at the base, not or slightly constricted at the septum, pedicels sub-hyaline, persistent, 47–96 × 12–19 μm (av. = 68 × 16 μm, n = 30), with a swollen base that gradually shows orange-yellow contents towards the lower end. Pedicel broken; paraphyses not seen.

**Figure 3.**
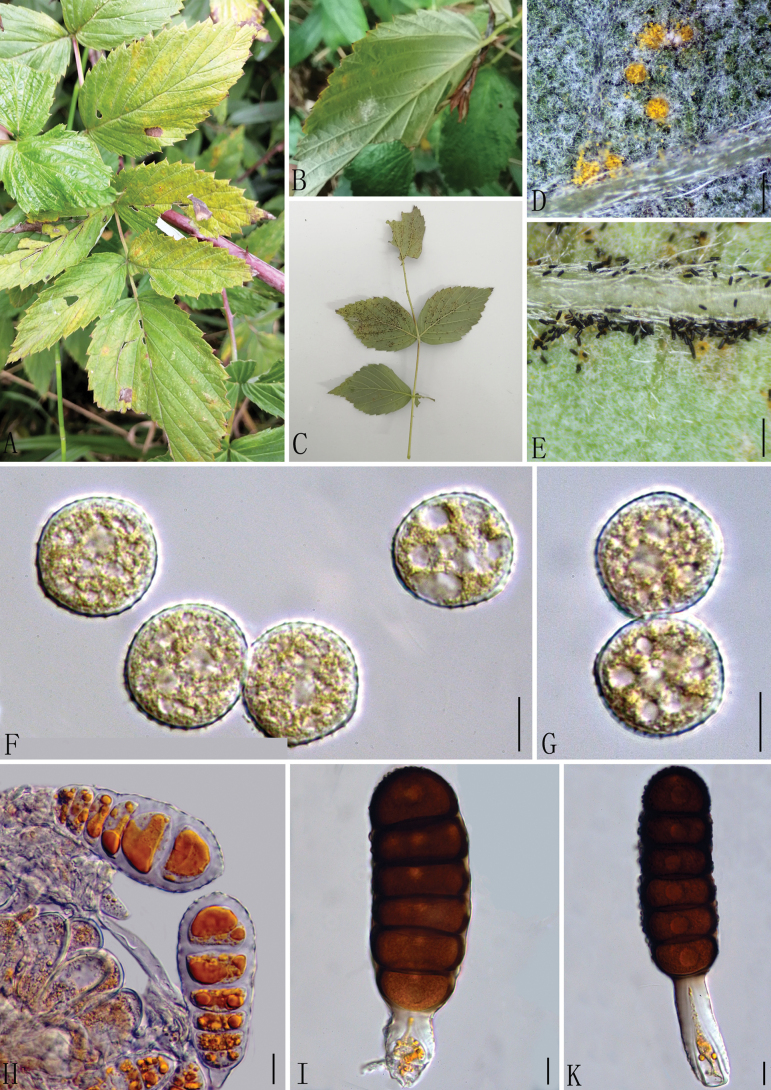
*Phragmidiumcoreanicola* (Holotype, GMB0101) **A–C** host and its habitat **D–E** uredinia and telia **F, G** urediniospores **H–K** teliospores. Scale bars: 0.5 mm **(D, E)**; 10 μm (**F–K)**.

###### Additional material examined.

China • Guizhou Province, Qingzhen City (26°34'58"N, 106°28'28"E), 1972 m a.s.l., on leaves of *Rubuscoreanus* (Rosaceae), 7 October 2021, Q.Z. Wu (GMB4071).

###### Notes.

*Phragmidiumcoreanicola* formed a separate branch in our phylogenetic analyses (Fig. 1). Morphologically, *P.coreanicola* differs from *P.griseum* in having slightly wider urediniospores (14–25 μm vs. 13–21 μm) and larger teliospores (107–167 × 25–35 μm vs. 50–125 × 18–28 μm) (Liu et al. 2018). Additionally, *P.coreanicola* is reported on *Rubuscoreanus*, whereas *P.griseum* was found on *Rubuscrataegifolius*. *Phragmidiumcoreanicola* differs from *P.cibanum*, which is reported on *Rubusniveus*, by having bigger urediniospores (20–29 × 14–25 μm vs. 17–20 × 18–19 μm) and larger teliospores (107–167 × 25–35 μm vs. 80–100 × 20–30 μm) (Wei 1988; Hiratsuka et al.1992; Liu et al. 2018). *Phragmidiumcoreanicola* has the same host species as *P.rubi-coreani* in Guiyang City. However, *P.coreanicola* has larger teliospores (107–167 × 25–35 μm vs. 29–74 ×14–37 µm) (Sun et al. 2022). The morphological comparison between *P.coreanicola* and *P.pauciloculare* shows that the *uredinia* of *P.coreanicola* are larger than those of *P.pauciloculare* (0.1–0.7 mm diam vs. 0.2–0.3 mm diam), and teliospores of *P.coreanicola* are also larger than those of *P.pauciloculare* (107–167 × 25–35 μm vs. 35–111 × 18–27 µm) (Wei 1988).

Furthermore, the ITS sequences of *P.coreanicola* and *P.griseum* exhibit significant differences, with a similarity of 87.33%. However, the LSU sequences of *P.coreanicola* and *P.griseum* have little variation, sharing a similarity of 99.38%.

##### ﻿ Phragmidium parvifolius

Taxon classificationFungiPuccinialesPhragmidiaceae

Q. F. Zhang, Q. Z. Wu & Q. R. Li sp. nov.

751ADE41-8F22-523A-8907-1B2CB804FA77

855006

Fig. 4

###### Type.

China • Guizhou Province, Guiyang City, Huaxi District (26°43′27.3″N, 106°67′14.4″E), 1,114 m a.s.l., on leaves of *Rubusparvifolius* (Rosaceae), 3 November 2022, Q. Z. Wu and Q. F. Zhang (holotype GMB4054, isotype KUN-HKAS144250).

###### Etymology.

The epithet refers to the host species, *Rubusparvifolius* L., from which the holotype was collected.

###### Description.

***Spermogonia***, ***Aecia*** and ***Telia*** not found. ***Uredinia*** 0.3–0.8 mm diam., produced on the abaxial leaf surface, scattered to gregarious, hypophyllous, rounded to irregular, powdery, orange, pulverulent, at first covered by the epidermis, later, not surrounded by host epidermis; Urediniospores 18–32 × 12–24 μm (av. = 22 × 18 μm, n = 30), globose, oblong, orange, wall 1.1–1.7 μm thick (av. = 1.3 μm, n = 30) at sides, regularly echinulate with stout spines; germ pores 2–3, supra-equatorial. Paraphyses 49–83 × 10–19 μm (av. = 65 × 15 μm, n = 30), hyaline, curved.

**Figure 4.**
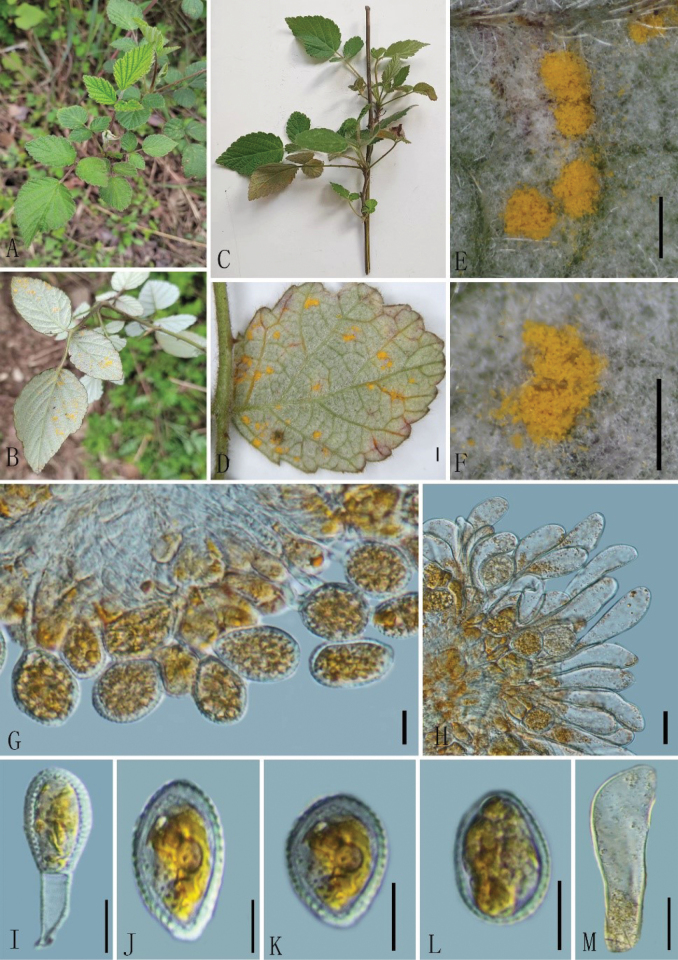
*Phragmidiumparvifolius* (Holotype, GMB4054) **A–D** host and its habitat **E, F** uredinia under a stereomicroscope **G, H** urediniospores and paraphyses **I–L** urediniospores **M** paraphyses. Scale bars: 1 mm (**D)**; 0.5 mm (**E, F)**; 10 μm (**G–M)**.

###### Additional material examined.

China • Guizhou Province, Guiyang City, Huaxi District (26°43′59.7″N, 106°67′66.5″E), 1114 m a.s.l., on leaves of *Rubusparvifolius* (Rosaceae), 3 November 2022, Q. Z. Wu and Q. F. Zhang (GMB4070).

###### Notes.

Phylogenetically, *P.parvifolius* formed a sister branch to *P.barnardii* Plowr. & G. Winter (HGU21035), which was also reported on *Rubusparvifolius* (Fig. 1). Morphologically, *P.parvifolius* can be easily differentiated from *P.barnardii* by its larger urediniospores (18–32 × 12–24 μm vs. 16–19 × 15–18 µm) and larger paraphyses (49–83 × 10–19 μm vs. 26–39 × 10–13 µm) (Winter 1886; McTaggart et al. 2016; Sun et al. 2022). In terms of urediniospore size, *P.parvifolius* is similar to *P.griseum* (Dietel) Syd. However, *P.parvifolius* differs from *P.griseum* by having relatively larger paraphyses (49–83 × 10–19 μm vs. 34–70 × 7–16 μm) and by its host, *Rubusparvifolius* vs. *Rubuscrataegifolius* (Wei 1988; Hiratsuka et al. 1992; Liu et al. 2018; Sun et al. 2024). Additionally, *P.parvifolius* differs from *P.pauciloculare* by its larger urediniospores (18–32 μm vs. 13–20 μm) (Wei 1988; Hiratsuka et al. 1992). *Phragmidiumparvifolius* and *P.kanas* have the same urediniospores and paraphyses, but *P.parvifolius* has no teliospores, whereas *P.kanas* has them (Zhao et al. 2021).

Furthermore, the morphological comparison between *P.parvifolius* and *P.coreanicola* (this study) shows that the urediniospores of *P.parvifolius* are larger than those of *P.coreanicola* (18–32 μm vs. 20–29 μm), and *P.parvifolius* has no teliospores, whereas *P.coreanicola* has them. The ITS and LSU sequence similarities of *P.parvifolius* with *P.coreanicola* are 97.57% and 99.22%.

##### ﻿ Gerwasia rubi-setchuenensis

Taxon classificationFungiPuccinialesPhragmidiaceae

J.E. Sun, Yong Wang bis & K.D. Hyde, (2024)

0B748040-F67A-5F99-A29D-23FAA12DCB04

Fig. 5

###### Host.

*Rubusbuergeri* Miq.

###### Description.

***Spermogonia***, ***aecia***, and ***telia*** unknown. ***Uredinia*** 0.4–1.0 mm diam., hypophyllous, pulverulent, golden, scattered, irregular, surrounded by host epidermis. *Urediniospores* 24–30 × 19–25 µm (av. = 27.2 × 22.3 µm, n = 30), subglobose or fusiform, inclusions golden or bright yellow; wall 1.4–2.9 µm thick (av. = 2.0 μm, n = 30), colorless, irregularly elongated verrucae.

###### Materials examined.

China • Guizhou Province, Zunyi City, Xishui County (28°49'38"N, 106°41'23"E), 1223 m a.s.l., on the leaves of *Rubusbuergeri* Miq. (Rosaceae), 3 November, 2022, Q. Z. Wu and Q. F. Zhang (GMB4052); China • Guizhou Province, Zunyi City, Xishui County (28°33'43"N, 106°24'3"E), 1997 m a.s.l., on *Rubusbuergeri* (Rosaceae), 3 November 2022, Q. Z. Wu and Q. F. Zhang (GMB4075).

**Figure 5.**
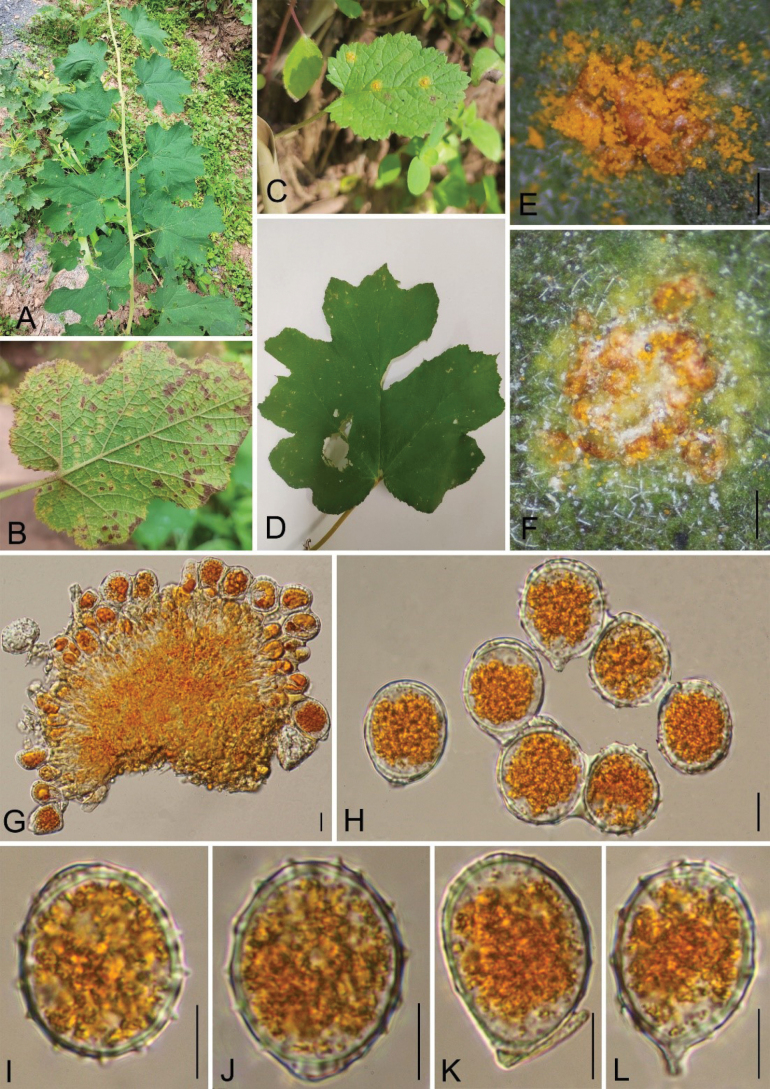
*Gerwasiarubi-setchuenensis* (GMB4052) **A–D** host and its habitat **E–F** uredinia under the stereomicroscope **G–K** urediniospores. Scale bars: 0.5 mm (**E–F)**; 10 μm (**G–L)**.

###### Notes.

In the phylogram (Fig. 1), our collections (GMB4052 and GMB4075) clustered with *G.rubi-setchuenensis* (HGUP21168). The morphological characteristics of our specimen are consistent with the original description of *G.rubi-setchuenensis*, and the DNA sequence aligns with that of *G.rubi-setchuenensis* HGUP21168 (ITS 100%; LSU 99.67%) (Sun et al. 2024). The only difference observed between the descriptions and figure of *G.rubi-setchuenensis* in Sun et al. (2024) is the size of the urediniospores. The urediniospores of *G.rubi-setchuenensis* (GMB4075) are slightly wider than those of *G.rubi-setchuenensis* (HGUP21168) (19–25 μm vs. 15–22 μm). This study identifies *Rubusbuergeri* as a new host for this fungus.

## ﻿Discussion

The exploration of rust fungi in China began in the mid-19^th^ century, and to date, over 1200 rust taxa have been documented (Zhuang et al. 1998, 2005, 2021; Zhao et al. 2022a, b; Sun et al. 2024). Molecular techniques have significantly advanced fungal species identification, but accurately identifying rust fungi remains challenging, necessitating a comprehensive approach incorporating morphology, host specificity, and phylogenetic analyses (Sun et al. 2024). In China, over 70 species of *Phragmidium* have been described, although numerous species remain without molecular data. According to our literature review, approximately 22 species of *Phragmidium* have been reported in Guizhou (Cummins 1931; Zhuang et al. 2012; Aime et al. 2018; Aime and McTaggart 2021; Zhao et al. 2021; Sun et al. 2022, 2024). These studies emphasize the critical role of integrating morphological data, host specificity, and phylogenetic insights for a comprehensive understanding and accurate identification of rust fungi.

In our investigation, three new species of Phragmidiaceae belonging to the genera *Gerwasia* and *Phragmidium* are introduced based on phylogenetic analysis of the ITS and LSU regions and morphological features. *amphidasydis* sp. nov., *Phragmidiumcoreanicola* sp. nov., and *P.parvifolius* sp. nov. infected *Rubusamphidasys*, *Rubuscoreanus*, and *Rubusparvifolius*, respectively. The host of the *P.coreanicola* is the same as that of *P.rubi-coreani*; however, *P.coreanicola* has larger teliospores (107–167 × 25–35 μm vs. 29–74 ×14–37 µm), and *P.rubi-coreani* possesses aeciospores (Sun et al. 2022). In addition, *Rubusbuergeri* was identified as a new host plant for *Gerwasiarubi-setchuenensis.* Previously, it was known only on *Rubussetchuenensis* (Sun et al. 2024). At the same time, we discovered some samples of *Phragmidiumrosae‐multiflorae* Dietel, and *Phragmidiumpotentillae* (Pers.) P. Karst. Here, we provide their sequences.

The hosts of *Phragmidium* discussed in this study are mainly from the genus *Rubus* within the Rosaceae family, yet *P.coreanicola* represents a new species on a previously reported host, while *Gerwasiarubi-setchuenensis* originates from a different host. This demonstrates the host specificity and species diversity of rust fungi (Wei 1988; Zhuang et al. 2012; Yang et al. 2015; Liu et al. 2018). Investigations into the interaction between plant hosts and pathogens suggest that the variety of pathogenic fungi may stem from host-switching or cooperative coevolutionary processes. These findings prompt inquiries concerning the linkage between plant hosts and *Phragmidium*, as well as the evolutionary dynamics at play (Zhao et al. 2016; McTaggart et al. 2015, 2016). Addressing these inquiries necessitates further research into *Phragmidium* in the future.

## Supplementary Material

XML Treatment for Gerwasia amphidasydis

XML Treatment for Phragmidium coreanicola

XML Treatment for Phragmidium parvifolius

XML Treatment for Gerwasia rubi-setchuenensis

## ﻿Additional information

### Author ORCIDs

Qinfang Zhang https://orcid.org/0009-0003-9408-4988

Kamran Habib https://orcid.org/0000-0003-2572-0306

Yao Wang https://orcid.org/0000-0002-1262-6700

Muhammad AIjaz Ahmad https://orcid.org/0000-0003-2798-5500

Yulin Ren https://orcid.org/0009-0003-9063-425X

Xiangchun Shen https://orcid.org/0000-0002-4333-9106

Qirui Li https://orcid.org/0000-0001-8735-2890

### Data availability

All the specimens are deposited in the Herbaria of Guizhou Medical University (GMB) and Kunming Institute of Botany, Chinese Academy of Sciences (KUN-HKAS). Sequences have been deposited in the GenBank. The alignment file can be obtained from the corresponding author.
